# Supplementary material for: K+ Channel Tetramerization Domain 5 (KCTD5) Protein Regulates Cell Migration, Focal Adhesion Dynamics and Spreading through Modulation of Ca2+ Signaling and Rac1 Activity
Source: Cells. 2020 Oct 12;9(10):2273. doi: 10.3390/cells9102273 (PMC7600296; doi:10.3390/cells9102273)
Supplement: Supplementary file 1 [file cells-09-02273-s001.zip › Supplementary Data/201002 Supplementary material.docx]

Supplementary material

K^+^ Channel Tetramerization Domain 5 (KCTD5) protein regulates cell migration, focal adhesion dynamics and spreading through modulation of Ca^2+^ signaling and Rac1 activity

**Jimena Canales, Pablo Cruz, Nicolás Díaz, Denise Riquelme, Elías Leiva-Salcedo, and**

**Oscar Cerda.**

1. Supplementary Materials and Methods

*1.1. MCF-7 cells culture and transfection*

MCF-7 breast cancer cells were culture in DMEM/F12 medium (Thermo Fisher Scientific, catalog #12500062) supplemented with 10% v/v Fetal Bovine Serum and antibiotics (10,000 U/mL penicillin, 10 ug/mL streptomycin). Cells were incubated at 37 ˚C in a humidified atmosphere and 5% CO_2_. For KCTD5 knockdown, MCF-7 cells were transiently transfected using the Lipofectamine LTX reagent with a plasmid encoding a shRNA^Scramble^ sequence as a control or with plasmids encoding an shRNA sequence against KCTD5 (shRNA^KCTD5^ #2). The KCTD5-knockdown assays were performed 72 hours post-transfection.

*1.2. MCF-7 cells migration.*

Transwell Boyden chamber migration assays were performed to evaluate the cell migration in MCF-7 cells, as was described in the Materials and Methods section. In parallel, the remaining cells were lysed, and the KCTD5 expression levels were evaluated by immunoblotting, as was described in the Materials and Methods section.

*1.3. Viability determination*

In parallel to migration assays, cells were seeded in 96-wells culture plate in serum-free RPMI 1640 medium or RPMI 1640 medium containing 10% v/v FBS, at a density of 6.0 x 10^3^ cells per well for each condition. After 18 hours, cells were washed two times with DPBS and then cell viability was measured using 3-(4,5-Dimethylthiazol-2-yl)-2,5-Diphenyltetrazolium Bromide (MTT) reagent (Thermo Fisher Scientific, catalog #M6494) following the manufacturer´s instructions.

*1.4. Proteasomal degradation inhibition*

Cells were incubated with the proteasome inhibitor MG-132 (Enzo Life Science, catalog #BML-PI102-0005) at concentration of 2 μM for 16 hours. DMSO was used as vehicle control. After treatment, cells were lysed, and proteins of interest were analyzed by immunoblotting as was described in Materials and Methods section.


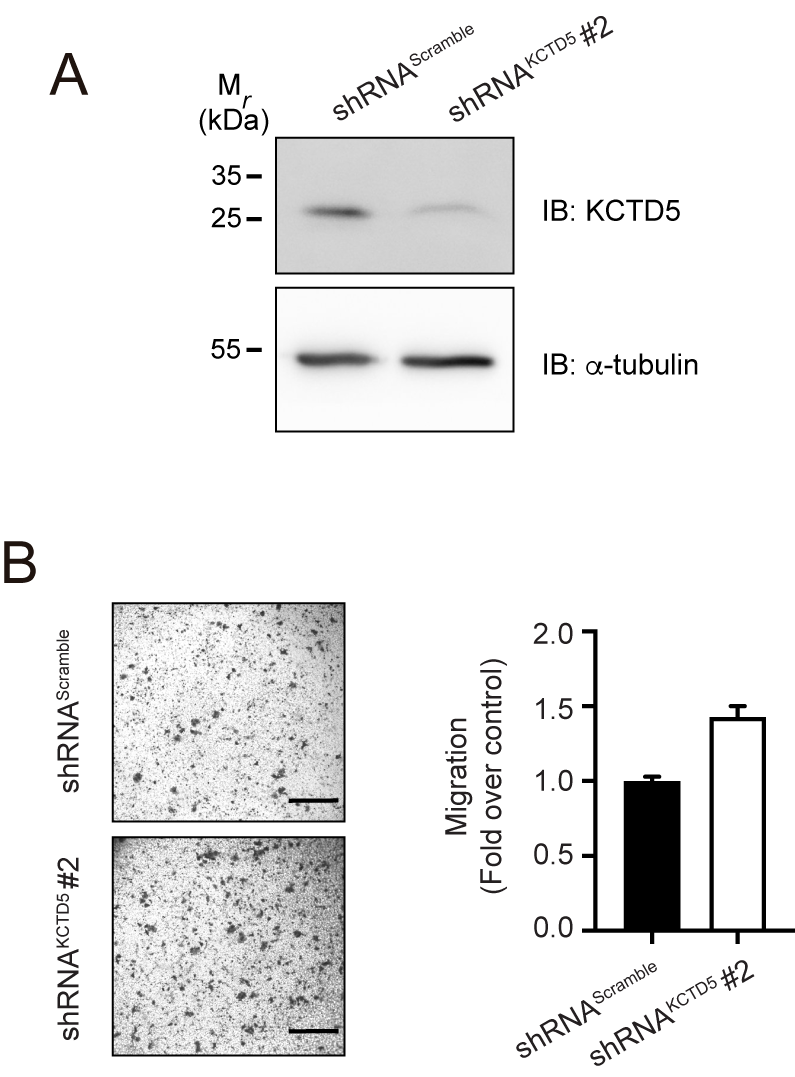


**Figure S1. KCTD5 silencing enhances MCF-7 cells migration.** A. Immunoblot showing the KCTD5 expression upon shRNA^Scramble^ and shRNA^KCTD5^#2 transfection in MCF-7 cells. α-tubulin was used as loading control. B. Transwell Boyden chamber migration assays of MCF-7 cells transfected with shRNA^Scramble^ or shRNA^KCTD5^#2 encoding plasmids. Cells were stimulated with 10 % v/v serum for 18 h. Scale bar=500 μm. Graph represents the relative migration for each condition (n=2)

**
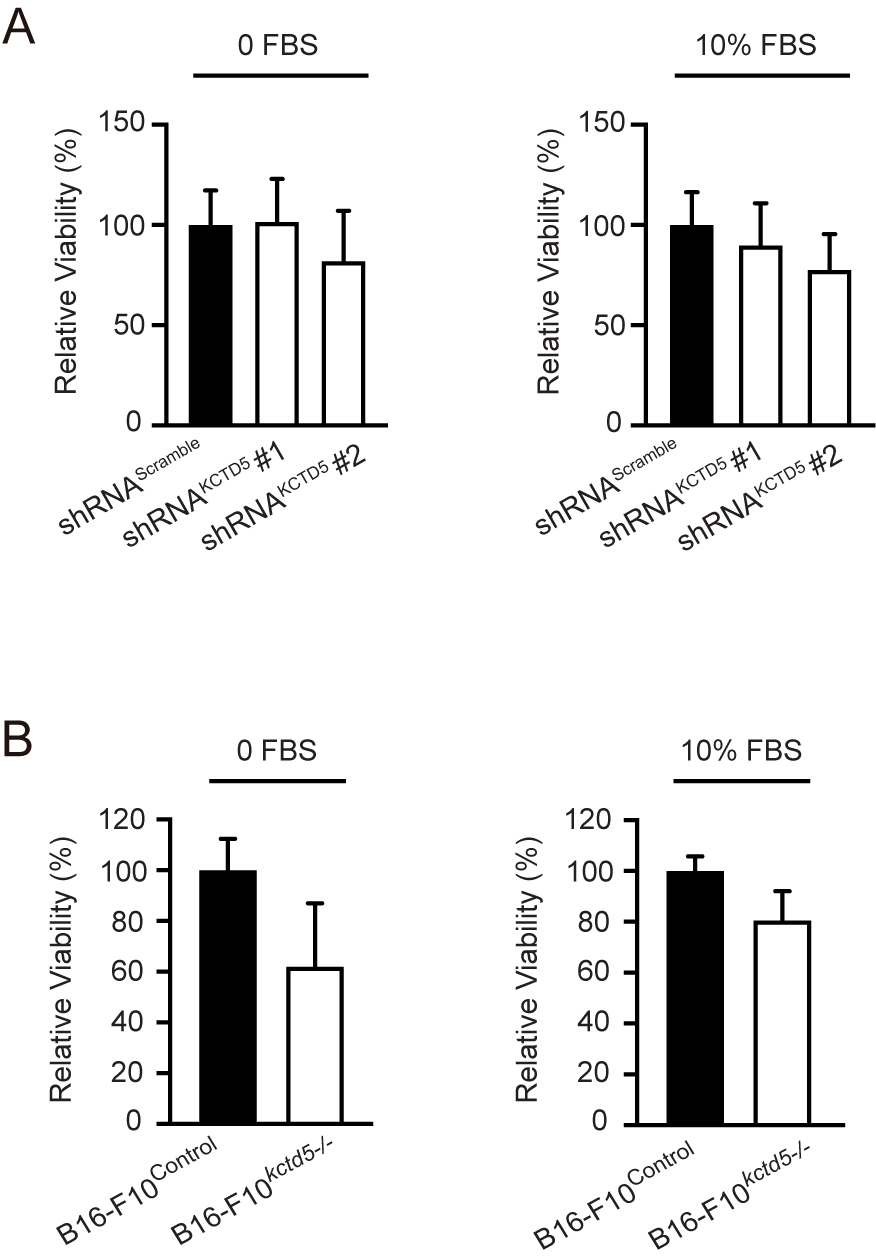
**

**Figure S2.** **KCTD5 does not affect the viability of B16-F10 cells.** A. Relative viability of B16-F10 cells transfected with shRNA^Scramble^, shRNA^KCTD5^ #1 or shRNA^KCTD5^#2 encoding plasmids. Cells were incubated in serum-free RPMI medium (left) or RPMI medium containing 10% v/v FBS (right) for 18 h. Then, viability was determined by MMT assay. Viability of control cells (shRNA^Scramble^) was considered as 100% for each case. (mean ± SD; n=4; One-way ANOVA followed by Tukey’s multiple comparisons test). B. Relative viability of B16-F10^Control^ and CRIPSR/Cas9-based KCTD5-Knockout B16-F10 cells (B16-F10*^kctd5-/-^*). Cells were incubated in serum-free RPMI medium (left) or in RPMI medium supplemented with 10% v/v FBS (right) for 18 h. Then, cell viability was determined using MTT reagent. Viability of B16-F10^Control^ cells was considered as 100% for each condition (mean ± SD; n=3; two-tailed unpaired Student’s t-test).


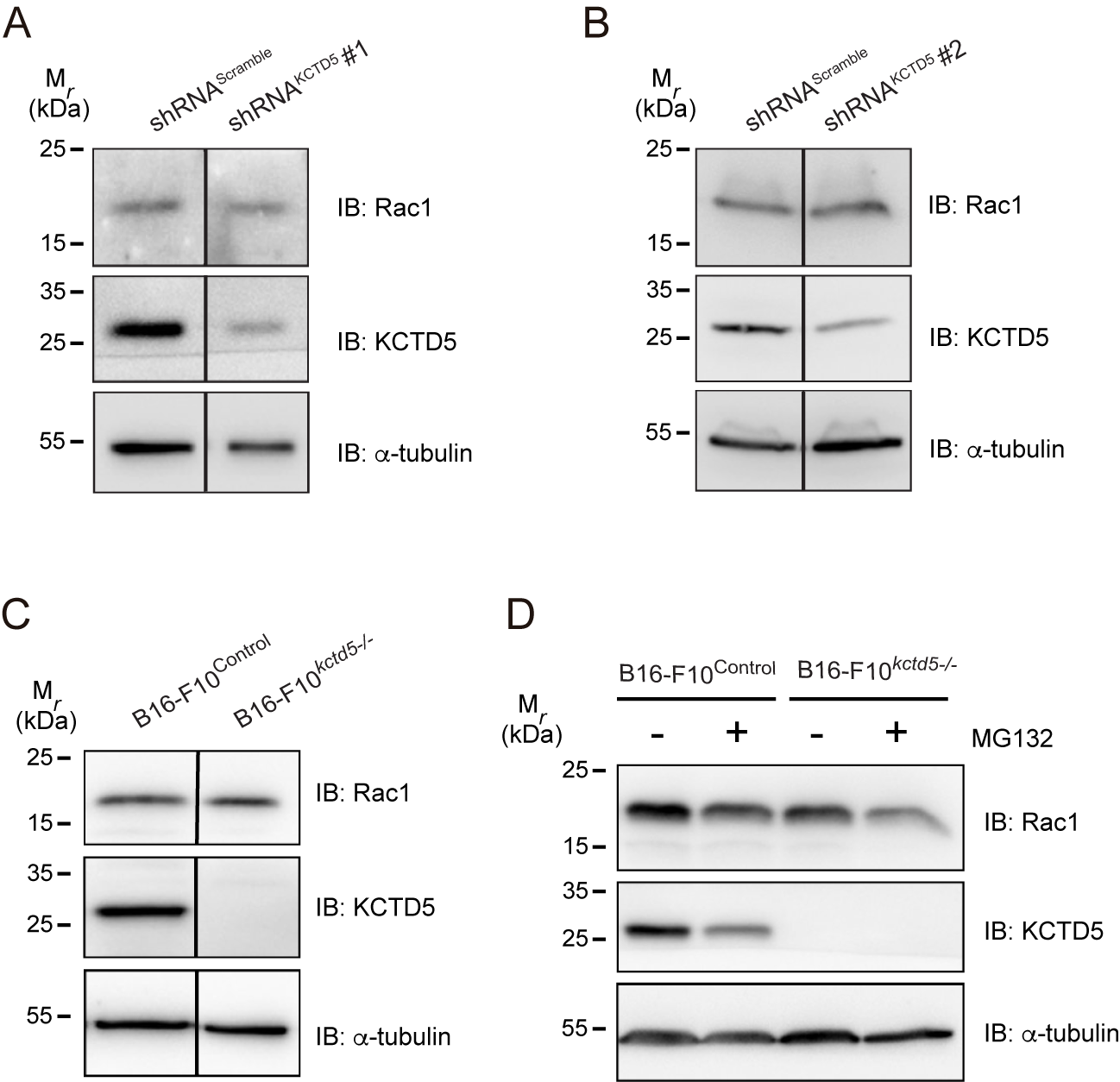


**Figure S3.** **KCTD5 is not involved in Rac1 degradation.** Immunoblots showing the Rac1 levels in B16-F10 cells transfected with shRNA^Scramble^ and shRNA^KCTD5^ #1 (A) or with shRNA^Scramble^ and shRNA^KCTD5^#2 (B) encoding plasmids. KCTD5 was analyzed to check the KCTD5-knockdown. α-tubulin was used as loading control. C. Analysis of Rac1 levels by immunoblot in B16-F10^Control^ and B16-F10*^kctd5-/-^*. KCTD5 was analyzed to check the KCTD5-knockout. α -tubulin was used as loading control. D. Immunoblot showing Rac1 levels in B16-F10^Control^ and B16-F10*^kctd5-/-^* cells treated with 2 μM MG-132 for 16 h. DMSO was used as vehicle control. KCTD5 was analyzed to check the KCTD5-knockout. α-tubulin was used as loading control.
